# Targeting Epstein-Barr Virus in Nasopharyngeal Carcinoma

**DOI:** 10.3389/fonc.2020.00600

**Published:** 2020-05-14

**Authors:** Pok Man Hau, Hong Lok Lung, Man Wu, Chi Man Tsang, Ka-Leung Wong, Nai Ki Mak, Kwok Wai Lo

**Affiliations:** ^1^Department of Anatomical & Cellular Pathology and State Key Laboratory of Translational Oncology, The Chinese University of Hong Kong, Hong Kong, China; ^2^Department of Biology, Hong Kong Baptist University, Hong Kong, China; ^3^Department of Chemistry, Hong Kong Baptist University, Hong Kong, China

**Keywords:** nasopharyngeal carcinoma, Epstein-Barr virus, EBNA1, cytolytic therapy, LMP1, BZLF1

## Abstract

Nasopharyngeal carcinoma (NPC) is consistently associated with Epstein-Barr virus (EBV) infection in regions in which it is endemic, including Southern China and Southeast Asia. The high mortality rates of NPC patients with advanced and recurrent disease highlight the urgent need for effective treatments. While recent genomic studies have revealed few druggable targets, the unique interaction between the EBV infection and host cells in NPC strongly implies that targeting EBV may be an efficient approach to cure this virus-associated cancer. Key features of EBV-associated NPC are the persistence of an episomal EBV genome and the requirement for multiple viral latent gene products to enable malignant transformation. Many translational studies have been conducted to exploit these unique features to develop pharmaceutical agents and therapeutic strategies that target EBV latent proteins and induce lytic reactivation in NPC. In particular, inhibitors of the EBV latent protein EBNA1 have been intensively explored, because of this protein's essential roles in maintaining EBV latency and viral genome replication in NPC cells. In addition, recent advances in chemical bioengineering are driving the development of therapeutic agents targeting the critical functional regions of EBNA1. Promising therapeutic effects of the resulting EBNA1-specific inhibitors have been shown in EBV-positive NPC tumors. The efficacy of multiple classes of EBV lytic inducers for NPC cytolytic therapy has also been long investigated. However, the lytic-induction efficiency of these compounds varies among different EBV-positive NPC models in a cell-context-dependent manner. In each tumor, NPC cells can evolve and acquire somatic changes to maintain EBV latency during cancer progression. Unfortunately, the poor understanding of the cellular mechanisms regulating EBV latency-to-lytic switching in NPC cells limits the clinical application of EBV cytolytic treatment. In this review, we discuss the potential approaches for improvement of the above-mentioned EBV-targeting strategies.

## Introduction

Nasopharyngeal carcinoma (NPC) is a malignant epithelial tumor affecting the lining of lymphocyte-rich nasopharyngeal mucosa. It is a distinct type of head and neck cancer, and its unique pathogenesis is influenced by multiple etiological factors such as genetic predisposition, diet, and Epstein-Barr virus (EBV) infection (1–3). Although NPC is rarely found in most parts of the world, it is prevalent in Southern China and Southeast Asia. There are up to 25 cases per 100,000 men in some parts of Southern China such as Zhong Shan City, Zhuhai, and Jiangmen. The high prevalence of NPC in these endemic regions implies that there are genetic and environmental factors that predispose individuals in these regions to develop this cancer (2, 3).

Notably, the strongest association with NPC risk has been consistently found in several variants of major histocompatibility complex (MHC) class I genes (4). Epidemiology studies have also documented dietary risk factors, such as consumption of salted fish or other preserved foods. The remarkable decrease of NPC incidence in some modern cities in endemic regions, such as Hong Kong, points to the effectiveness of changes in dietary habits for reducing exposure to these potential carcinogens (2, 5). Unlike other head and neck squamous carcinomas (HNSCCs), the major histological type of NPC is non-keratinizing carcinoma, either poorly or undifferentiated, showing characteristic features of rich lymphocytic infiltration and EBV infection. The link between EBV and NPC has been well-established by the fact that EBV DNA or transcripts are invariably detected in tumor cells, as well as the presence of a clonal EBV genome in NPC and precancerous lesions (6, 7). Next-generation sequencing-based studies have recently revealed that certain prevalent EBV strains are associated with an increased risk of NPC in Southern Chinese (8, 9). Strikingly, a link was found between EBV viral genomic variation and reportedly NPC-susceptible single nucleotide polymorphisms (SNPs) at the HLA locus (9). This new finding suggests that there is a complex interaction of genetic and viral factors involved in the pathogenesis of NPC.

The therapeutic management of NPC is based on the disease stage, according to the National Comprehensive Cancer Network guidelines (v. 2.2018). Radiotherapy (RT) alone is the major therapeutic strategy to manage early-stage disease (Stage I); RT in combination with concurrent chemotherapy (CRT) is used to manage intermediate (Stage II) to advanced stages of NPC (Stages III-IV) (2). According to a clinical study in Hong Kong, patients with early-stage NPC have favorable clinical outcomes and their survival rates with standalone RT are encouraging, with a 5-year overall survival rate of ~90% (10). The adoption of intensity-modulated radiotherapy (IMRT) combined with conformal radiotherapy, which improves the diametric properties and reduces the toxicity of irradiative treatment, further significantly improves the locoregional control of NPC and the overall survival rate of patients (2, 11).

Nevertheless, >60% of newly diagnosed patients have a poor clinical outcome, as they usually present with advanced-stage disease. Most NPC patients will later develop loco-regional (5–15%) and distant treatment failures (15–30%) (2, 12). Furthermore, half of the patients with local recurrence also experience concurrent distant metastasis. In addition, ~30% of patients with late-stage disease will experience distant recurrence following intensive concurrent CRT (2, 13).

Currently, the treatment of recurrent and metastatic NPC is challenging, and the clinical outcomes remain uncertain, possibly due to the profound heterogeneity of patients. Recent clinical studies of various new treatment strategies such as palliative systemic chemotherapy (e.g., gemcitabine plus cisplatin), targeted molecular therapies (e.g., VEGFR and EGFR inhibitors), and immunotherapies (e.g., adaptive T-cell therapy and immune checkpoint blockades) for controlling the progression of disease have shown a range of success rates (2, 12–14). Although the genomic landscape of NPC has recently been defined, disappointingly only a subset of NPC cases (>10%) was found to harbor immediately druggable somatic events, such as alterations of PIK3CA, FGFR3, and JAK1/2 (3, 15, 16). Moreover, the clinical benefits of the approved drugs targeting these potential oncogenic mutations still need to be confirmed in these patients. The discovery of frequent somatic alterations of MHC class I molecules also suggests that most NPC patients develop resistance to T-cell-based immunotherapy (3, 15). Thus, it is vital that effective therapeutic strategies are developed to address the unique features and specific molecular targets of NPC, to enable eradication of this deadly disease.

## EBV Infection in NPC

EBV, also known as human gammaherpesvirus 4, is a double-stranded DNA virus with a 170–180 kb genome that encodes nearly 100 genes for either latent or lytic infection of host cells. During the latent phase of infection, the viral genome remains episomal and expresses a group of latent genes (>10) for modulating various cellular mechanisms and exploiting host DNA polymerases for DNA replication. In contrast, lytic infection results in the expression of >80 lytic proteins and the extracellular release of viral particles during mandatory cell death.

Globally, over 90% of adults are healthy carriers of lifelong EBV infection, although the virus is now classified as a group I carcinogen. In healthy carriers, primary infection is followed by the persistence of EBV latency in only a few memory B-cells, and is under the control of the host's immune system. Nevertheless, the virus contributes to tumor initiation and clonal expansion of infected lymphoid and epithelial cells by inducing specific genetic/epigenetic changes (such as c-myc translocation and loss of *CDKN2A/p16*) or impairing host immune system (6, 17).

EBV is the first oncogenic virus identified in human cancer, and is etiologically linked to a remarkably wide range of human lymphoid malignancies (such as Burkitt lymphoma, classic Hodgkin lymphoma, B-cell lymphoma, and nasal NK/T-cell lymphoma), two distinct types of epithelial cancer, gastric cancer (GC), and NPC. Among the 200,000 new cases of EBV-associated cancers reported annually worldwide, 84,000 and 78,000 are GC and NPC, respectively. Nevertheless, EBV-associated GCs represent only ~10% of all gastric cancers and are not endemic. In the endemic regions, such as Hong Kong and Southern China, almost all NPCs are of the non-keratinizing subtype, which is consistently associated with EBV infection (17, 18).

For the past three decades, studies have revealed that NPC tumorigenesis is driven by EBV infection and a combination of multiple genetic aberrations. It is believed that NPC is a clonal malignancy derived from a single progenitor cell that was latently infected with EBV (1, 3, 7). This is evidenced by the fact that all episomal EBV genomes within NPC cells contain the same number of terminal repeats (TRs), which can only result from latent replication of EBV from one progenitor cell. However, in the EBV lytic cycle and its subsequent infection of epithelial cells, the linearized EBV genomes from the infectious virions undergo circularization by random joining of the TRs to form episomes, resulting in various numbers of TRs being present in each EBV episome within the latently infected cells (19, 20).

In an early study by Pathmanathan et al. (20), both EBV latent gene products (e.g., *EBERs* and LMP1) and homogeneous lengths of TR repeats were detected in NPC and precancerous lesions, suggesting that the clonal latent EBV infection is a crucial event in the initiation of this virus-associated cancer (20). Furthermore, our earlier genomic and functional studies have indicated that several specific genetic alterations (such as inactivation of *CDNK2A/p16* and tumor suppressors at chromosome 3p) in the premalignant nasopharyngeal epithelium support a cellular switch to state that maintains persistent latent EBV infection and predisposes individuals to NPC transformation (21–23). Indeed, persistent EBV latent infection and expression of latent viral genes are essential for NPC development. A type II latency program is observed in NPC, in which *EBER1/2*, EBNA1, LMP1, LMP2, BARF1, and multiple splicing non-coding RNAs and a number of miRNAs in *BART* regions are expressed. Several latent genes, such as *LMP1* and *LMP2*, are heterogeneously expressed in the tumor or during progression, while *EBERs* and *EBNA1* are consistently detected in all cancer cells (6, 18).

Notably, although loss of the EBV genome has been reported during long-term passage of some NPC cell lines *in vitro*, latent EBV infection is consistently detected in every tumor cell in patient-derived xenograft (PDX) models and clinical NPC specimens, in both primary or recurrent cases (6, 18, 24, 25). The continued presence of an episomal EBV genome and the requirement of multiple viral gene products for malignant transformation have been shown as key features of EBV-associated NPC.

Studies have also shown that multiple viral latent genes contribute to NPC tumorigenesis by generating various hallmarks of cancer. The oncogenic properties of these latent gene products and their contribution to NPC tumorigenesis have been extensively studied in epithelial cell lines over the past three decades (6, 18). Among these latent gene products, EBNA1 is the only protein that is expressed in all of the EBV-associated cancers: it is essential for governing the replication and mitotic segregation of the EBV episomes, thereby maintaining EBV genomes in latently infected cells. In addition, there is emerging evidence that EBNA1 plays roles in promoting cell survival upon DNA damage, inducing genetic instability and transcriptionally activating various cellular genes (26).

In addition to EBNA1, abundant non-polyadenylated RNAs, such as *EBER1* and *EBER2*, are also detected in all EBV-positive cancer cells. In latent infected epithelial cells, *EBERs* bind to auto-antigen La and ribosomal protein L22 to form ribonucleoprotein particles. This complex then binds to the PKR to prevent Fas-mediated apoptosis (27). Furthermore, these non-coding RNAs were also shown to promote tumor growth by stimulating secretion of autocrine insulin-like growth factor (IGF-1) and activating the NF-κB pathway via retinoic acid-inducible gene-1 (RIG-1) and toll-like receptor 3 (TLR3) signaling (28–30).

In NPC cells, multispliced long non-coding transcripts and viral miRNAs from the *BamH1 A* region of the EBV genome are abundantly expressed. As described in recent reviews, EBV-encoded miRNAs, *miR-BARTs*, target multiple viral and cellular genes to facilitate EBV latency, promote cell proliferation, enhance invasiveness, induce genome instability, inhibit apoptosis, and impair host immune response (6, 31, 32). Recent studies have also revealed that long non-coding RNAs (e.g., *RPMS1*) may epigenetically regulate cellular gene expression and maintain EBV latency by interfering with chromatin remodeling machinery, subsequently contributing to NPC tumorigenesis (33, 34). A BARF1 protein encoded by the *Bam H1-A* fragment is a homolog of human colony-stimulating factor 1 (CSF1) receptor, and this secreted viral protein is believed to enhance NPC tumorigenicity through activation of the CSF-1 signaling axis, suppression of apoptosis by activation of BCL-2, and upregulation of expression of NF-κB, RelA, and cyclin D1 (35).

LMP1 is a key EBV-encoded oncoprotein that functions as a potent activator of multiple signaling cascades, such as NF-κB, MAPK, JNK/AP1, and PI3K, to generate multiple cancer hallmarks (7, 36). Although LMP1 is only highly expressed in a subset of NPC specimens, the occurrence of LMP1 in preinvasive lesions implicates its contribution in transforming nasopharyngeal epithelial cells and tumor initiation (15, 20). LMP1 may enhance self-renewal properties and thus promote a cancer progenitor-like cell phenotype in a subpopulation of cancer cells, thereby driving the progression of NPC (36–38). LMP2A is another integral membrane protein that promotes stem-like properties and various oncogenic phenotypes by regulating multiple signaling pathways, such as PI3K/AKT, ERK, and RhoA (36, 38, 39). Unlike LMP2A, the function of LMP2B, which is encoded by an alternative first exon of the LMP2 gene, remains unclear.

Given the above oncogenic properties of EBV latent gene products and the unique virus-cell interactions, targeting these latent proteins and inducing lytic reactivation are thought to be possible approaches to cure this viral-associated epithelial cancer.

## Targeting EBV Latent Proteins

The viral-encoded latent proteins EBNA1, LMP1, and LMP2 are expected to be potential therapeutic targets in NPC cells. The function of EBNA1 has been intensively studied because of its consistent expression in every tumor cell and its essential role in the maintenance of the EBV episomal genome. Indeed, the consistent expression and the biological importance of EBNA1 in viral DNA maintenance, replication, and segregation during viral latency and lytic reactivation make the EBNA1 protein a key therapeutic target. Research efforts over the past decade indicate that EBNA1 is a druggable protein, and selective agents targeting the DNA-binding site or dimerization interface have demonstrated efficacy in animals. The protein sequence of EBNA1 has little similarity to the cellular protein of the host, except the reported similarities between the EBNA1 epitopes (PPPGMRPP and (GR)x) and the common human antigenic targets of the lupus autoantigens (Sm B'(PPPGMRPP) and Sm D1 (GR)x) (40). Nevertheless, it is expected that the off-target effect of a well-designed EBNA1-targeting agent would be minimal.

### Therapeutic Targeting of EBNA1

**Figure 1:**
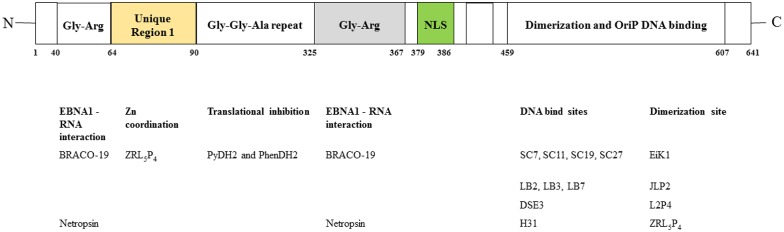
Functional regions in EBNA1. **(Upper)** Domains of EBNA1. NLS, nuclear localization sequence. **(Lower)** Agents reported to inhibit these domains. Refer to text for details.

**Figure 2:**
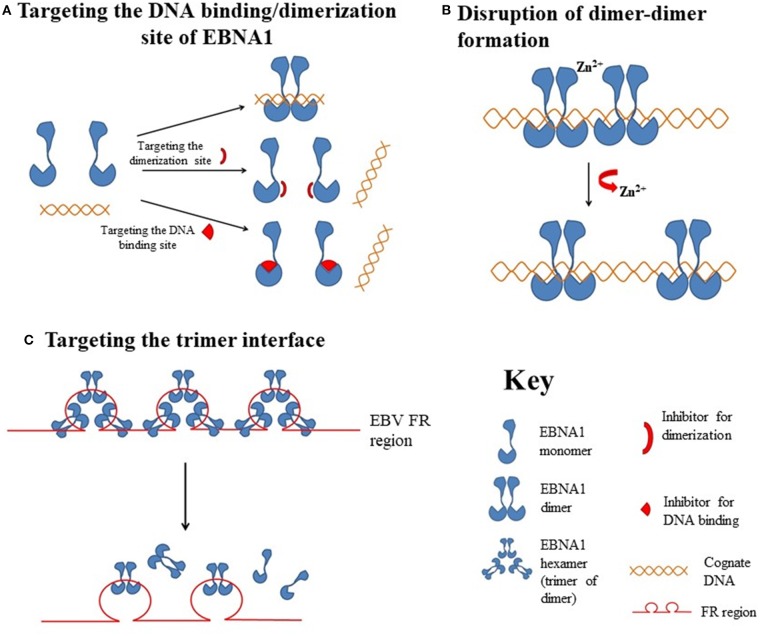
Hypothetical diagrams of the EBNA1-targetable sites for therapeutic intervention. **(A)** Targeting the DNA binding or dimerization site of EBNA1. **(B)** Disruption of the EBNA1 dimer-dimer formation. **(C)** Targeting of the EBNA1 trimer interface.

It is now clear that EBNA1 interacts with certain host cell components to establish viral latency and mediate oncogenic transformation of the host cells (26, 41). EBNA1 is also considered to be a unique episome maintenance protein (42); several regions contributing to these processes have been identified (Figure 1). Early studies showed that EBNA1 siRNA could inhibit the growth of EBV-positive epithelial tumors and increase lytic DNA replication (43, 44). With the recent advances in the understanding of the structural biology of EBNA1, emerging evidence indicates that both EBNA1 dimers and oligomers participate in the control of viral latency. Here, we review the approaches that have been examined for the disruption of EBNA1 functions and the feasibility of targeting EBNA1 for treatment EBV-associated diseases (Figure 2, Table 1).

**Table 1:** Therapeutic targeting of EBNA1 dimerization or multimerization.

**Proposed targeting site**	**Remarks**	**References**
DNA-binding site	•Molecular docking interaction analysis of compounds SC7 and SC19 identified several crucial residues such as Arg469 and Tyr518 of EBNA1	(45)
DNA-binding site	•Fragment-based approach and X-ray crystallography •A 2,3-disubstituted benzoic acid series that selectively inhibits the DNA-binding activity of EBNA1 •Suppresses tumor growth *in vivo*	(46)
Dimerization site	•Interruption by an engineered peptide _561_YFMVF_565_ with NLS RrRK. RrRK forms a salt with D_602_ •Suppresses tumor growth *in vivo*	(47)
Dimerization site	•A Zn^2+^ chelator conjugated with EBNA1-binding _561_YFMVF_565_ and NLS RrRK peptide •Inhibits EBNA1 oligomerization, suppresses tumor growth *in vivo*	(48)
Dimer-dimer interface	•At DS half-site •H-bond network involving residues R491 and D581	(49)
Dimer-dimer interface coordinated by Zn	•*In silico* protein structure modeling •Two conserved cysteine residues (Cys79 and Cys82) coordinate Zn^2+^ and facilitate the multimeric interactions	(50)
Interface between dimer and hexamer (trimer of dimers)	•EBNA1 forms hexamer at FR and hexamers stack to form an array of multiple hexagonal wheel. Essential for the maintenance of episome and latent infection •T585 is critical for the H-bonding network with adjacent residues as well as with residues from adjacent EBNA1 molecules	(51)

*NLS, nuclear localization sequence; DS, dyad symmetry; FR, family of repeats*.

#### Interfere the RNA-Binding Function of LR1 and LR2 Regions in the EBNA1 Protein

The linking regions LR1 (amino acids 40–89, also known as GR1) and LR2 (amino acids 325–379, also known as GR2) are arginine- and glycine-rich regions that resemble the RGG motifs for RNA binding. It has previously been demonstrated that EBNA1 recruits the cellular origin recognition complex (ORC) to origin of replication (oriP) for episome maintenance or replication initiation (52). The recruitment of ORC to the dyad symmetry (DS) of oriP occurs through an RNA-dependent interaction with the RGG-like motifs in LR1 and LR2 (53). A subsequent study showed that LR1 and LR2 can bind to G-quadruplex-structured G-rich RNA (54). BRACO-19 is a G-quadruplex-interactive molecule. Norseen and coworkers further demonstrated that BRACO-19 could disrupt the tethering of EBNA1 to the metaphase chromosomes, indicating that G-quadruplex-interactive molecules may be developed as inhibitors of the LR1/LR2-dependent viral DNA attachment and replication in EBV-infected cells.

#### Interfere the Binding of LR1 and LR2 Regions Containing AT-Rich DNA

In addition to RNA binding, both the LR1 and LR2 regions have been shown to contain an AT-hook DNA-binding domain for the tethering of EBNA1 to chromosomal DNA. In LR1, the domain ATH1 (amino acids 40–54) resembles the AT-hook of high mobility group A (HMGA) protein (55). Sears and coworkers previously demonstrated that the ability of EBV to stably replicate and partition oriP plasmids correlates with the AT hook activity of EBNA1 (56). Chakravorty and Sugden further demonstrated that a small molecule, netropsin, not only inhibits the AT-hook DNA-binding activity of EBNA1 *in vitro* but also forces the loss of EBV genomes in an AT-hook dependent manner in epithelial and lymphoid cells (57). The results from these studies suggested that small molecule-based pharmacological blockade of AT-hook activity may effectively perturb the viral latency in EBV-infected cancer cells.

#### Target the Glycine-Alanine Repeats (GAr) Region of EBNA1 mRNA

The ability of EBV-latently infected cells to evade immune recognition is attributed to the presence of glycine-alanine repeats (GAr) in EBNA1. GAr (amino acids 90–325) is located near the N-terminal of EBNA1. Previous studies indicated that nascent GAr has the capacity to suppress the translation of its own mRNA *in cis* (58). The suppression of initiation of mRNA translation by GAr can also prevent the presentation of antigenic fragments generated from EBNA1 by class I MHC molecules (59). Hence, GAr-based reduction of translation of EBNA1 has been implicated as a relevant therapeutic target in EBV-latently infected cells.

Using a yeast-based genetic screening assay, Lista and coworkers found that nucleolin can directly interact with G-quadruplexes formed in GAr-encoding *EBNA1* mRNA. Furthermore, the process of GAr-mediated inhibition of EBNA1 expression and antigen presentation can be reversed by blocking the binding of nucleolin to *EBNA1* mRNA with G-quadruplex ligand PhenDC3 (60). Subsequently, a series of cationic bis(acylhydrazone) derivatives, representing shape analogs of PhenDC3, were synthesized and tested. Two compounds, PyDH2 and PhenDH2, were found to enhance the expression of EBNA1 in H1299 cells in a GAr-dependent manner (61). The results from these studies indicated that interruption of the interaction between nucleolin and GAr of EBNA1 mRNA for the restoration of immune recognition of infected cells may be a therapeutic strategy for the treatment of NPC.

#### Target the DNA Binding/Dimerization Site of EBNA1

The DNA-binding domain of EBNA1 is located between amino acids 459 and 598, near its C-terminal (62, 63). Under native conditions, EBNA1 exists as a dimer (64), and EBV replication and EBNA1 transactivation depend on the formation of an EBNA1 dimer and the binding of the dimer to EBV DNA. Crystallographic studies of the DNA-binding region of the EBNA1 protein revealed that this region has two structural domains: a core domain for EBNA1 dimerization and sequence-specific DNA interaction, and a flanking domain for DNA contact (45).

In early studies using a high-throughput virtual screen of 90,000 low molecular weight compounds, Li and coworkers demonstrated that a series of four compounds (SC7, SC11, SC19, and SC27) with an IC50 of ~20 μM could physically inhibit EBNA1-DNA binding and reduce the number of EBV episomes in latently infected cells (65). Using a fluorescence polarization-based EBNA1/DNA binding high-throughput screening method, Thompson and coworkers, from the same group, identified several small-molecule inhibitors (LB2, LB3, and LB7 and LC7) from a library of 14,000 molecules that could selectively inhibit the binding of EBNA1 to DNA (46). In further studies using a fragment-based approach and X-ray crystallography, Messick and coworkers developed a series of 2,3-disubstituted benzoic acids that could selectively inhibit the DNA binding activity of EBNA1, and also suppress the growth of EBV-positive tumors in xenograft models (66). One of the inhibitors, VK-2019, is now in Phase I/IIa clinical trial (NCT03682055) in patients with EBV-positive NPC.

Most protein-protein interfaces are relatively flat, which means these sites are difficult to target with small-molecule drugs. Nonetheless, various protein-protein interfaces, such as dimerization interfaces, have emerged as a class of druggable targets (67–69). In EBNA1, interaction between dimerization interfaces is essential not only for the formation of EBNA1 dimer but also for the subsequent formation of the dimer-DNA complex, which makes the dimerization interface an attractive therapeutic target. A previous study showed that a short EBNA1 peptide (P85) covering amino acids 560–574 could effectively inhibit homodimerization of EBNA1 (47), and a peptide with a sequence of V_560_CYFMVFL_566_Q could substantially inhibit EBNA1- and oriP-dependent transcription of SEAP (secreted embryonic alkaline phosphatase) reporter in cells in a dose-dependent manner. Regarding the development of inhibitors for the dimerization interface, Jiang and coworkers demonstrated that a chemical probe consisting of nuclear localization sequence RrRK and the YFMVF motif could reduce the *in vitro* formation of EBNA1 dimer and inhibit *in vivo* growth of EBV-positive NPC (49).

#### EBNA1 Oligomers as Potential Targets

In addition to perturbing the functions of the EBNA1 dimer, interfering the formation of EBNA1 oligomers is another potential approach for disturbing EBV latency. The presence of two functionally distinct oligomeric states of EBNA1, namely a dimer-dimer and a trimer of dimers (hexamer), has recently been reviewed (42). According to the crystal structure of two “EBNA1 DNA-binding domain dimers” binding to a DS half-site, Lieberman and coworkers found that the dimer-dimer interface involves amino acids R491 and D581 and a hydrogen-bonding (H-bonding) network (70). Disruption of this interface could destabilize the formation of the dimer-dimer complex on the EBV DNA and subsequently impair the recruitment of MCM2 complex to oriP. The results from that study indicated that this dimer-dimer interface may be druggable by EBNA1-specific targeted therapeutic.

An early study showed that the unique region 1 (UR1) dimerizes upon coordinating with a zinc ion (Zn^2+^) through a pair of essential cysteines within this region, and disruption of the zinc coordination prevented self-association and EBNA1-dependent transcriptional activity (50). In a computational study, full-length EBNA1 was used to develop monomeric and dimeric models (51). Hussain et al. found that adjacent dimers could link through Zn^2+^, and that the bonding of Zn^2+^ with the N-terminal cysteines would facilitate the multimerization of the EBNA1 dimers. The results from these studies suggested that disruption of Zn^2+^ coordination might be an approach to prevent the oligomerization and the subsequent functions of EBNA1.

Apart from the formation of dimer-dimer complexes, the results from X-ray crystallographic studies show that EBNA1 may form a higher-order complex, namely a hexamer (trimer of dimers), as mentioned above (71). The formation of a hexameric structure at the family of repeats (FR) of oriP appears to be essential for the long-term maintenance of the EBV episomes. Among the trimer interface residues, namely R496, Q530, L582, M584, and T585, the last appears to be critical for the H-bonding network with both adjacent residues and adjacent EBNA1. As a T585 polymorphism is frequently found in Burkitt lymphoma and NPC, and the trimer interface is important for the maintenance of EBNA1 hexamer, this recently discovered interface may be another novel target for the disruption of the biological functions of EBNA1 multimers (72).

#### Development of EBNA1-Based Theranostic Agents

Theranostics is an innovative treatment modality that combines both diagnosis and targeted therapy, in the form of a single theranostic agent. Addition of an imaging moiety to the molecularly targeted agent would greatly facilitate the monitoring of the drug inside the cells or animal models. As mentioned above, EBNA1 is the only viral protein expressed in all EBV-infected cells, despite the existence of different latency types. The homodimerization of EBNA1 is known to be critical for EBNA1 to carry out all its major functions, such as viral DNA replication, segregation and maintenance of the EBV genome, and transcriptional activation/repression. In an early study, two types of inhibitors (peptide inhibitor P85, and small chemical inhibitor Eik1) were designed to target the DNA-binding/dimerization domain of EBNA1 (47). P85 contains a short EBNA1-derived β3 sheet (amino acids 560–566) that can target the region of the EBNA1 dimerization domain (amino acids 560–574). These EBNA1 inhibitors cannot be visualized inside the cells, and have low bioavailability due to their poor water solubility; to overcome this, we constructed a novel hybrid system containing a charged, water-soluble chromophore and an EBNA1-specific binding peptide **P**_**2**_, which was derived from the Y_561_FMVF_565_ amino acid residues of EBNA1 (73). This water-soluble chromophore-peptide bio-conjugate, **JLP**_**2**_, enables both simultaneous imaging and inhibition of EBNA1 *in vitro* in EBV-infected tumor cells. **JLP**_**2**_is the first generation of our EBNA1 dual-function bioprobes, and its cellular uptake can be evaluated directly by fluorescence detection; this is likely due to its slightly enhanced emission when bound to EBNA1, which disrupts EBNA1 formation, as indicated by cell-free assays. However, **JLP**_**2**_ lacks a specific subcellular location and is unable to penetrate the nucleus, and also does not show a significant responsive-binding fluorescent signal, which limits its further utility as an EBV-specific inhibitor.

#### A Nuclear Localizing EBNA1-Based Theranostic Agent: L_2_P_4_

Our subsequent study solved the problem of targeting the nuclear EBNA1 protein by incorporating a nuclear localization sequence (NLS) of the amino acid residues RrRK into the C-terminus of the penta-peptide **P**_**2**_(YFMVF). The resulting **P**_**4**_ (YFMVF-GG-RrRK) can occupy the first EBNA1 dimerization interface within the DNA-binding domain DBD (48, 49). The NLS sequence in **P**_**4**_ can form salt bridges with the adjacent dimerization interface, including several residues in the aspartate-rich tail of EBNA1 (D_602_, D_601_, D_605_), which further enhances the interaction between **P**_**4**_and the EBNA1 monomer.

The fluorophore **L**_**2**_ was coupled with **P**_**4**_ to form the second-generation EBNA1 bioprobe **L**_**2**_**P**_**4**_, which generates a responsive fluorescent signal when it binds with EBNA1 via induction of intermolecular charge transfer (ICT) in the **L2** fluorophore molecule. Confocal live-cell imaging clearly showed that the presence of NLS in **L**_**2**_**P**_**4**_ enabled its penetration into the nuclei of EBV-positive cells, but not EBV-negative cells. **L**_**2**_**P**_**4**_ can also significantly interfere with the EBNA1 dimerization, and it only inhibits the *in vitro* tumor-cell growth (leading to *in vivo* tumor suppression) of EBV-infected cells, and not of EBV-negative cells. The therapeutic potential of **L**_**2**_**P**_**4**_ in EBV-associated malignancies is therefore evident.

#### L_2_P_4_-Based Lanthanide Upconversion Nanoparticles

To further enhance the stability of the EBNA1-binding peptide **P**_**4**_, to prolong its fluorescent lifetime and to minimize interference by biological autofluorescence, **P**_**4**_was conjugated with the lanthanide upconversion nanoparticles (UCNPs) NaGdF_4_:Yb^3+^ and Er^3+^@NaGdF_4_ to form **UCNP-P**_**4**_ (74). Lanthanide-mediated upconversion is a well-known photophysical phenomenon characterized by the generation of high-energy photon/emission from low-energy photon/excitations. The **P**_**4**_peptide gained improved stability and biocompatibility from the solid support of UCNPs, which are quenched by the coating of **P**_**4**_ molecules, thus inducing aggregation of the UCNPs upon physical interaction with the EBNA1 protein molecules. This unique mechanism results in responsive UCNP emission and improves the signal-to-noise ratio for imaging purposes, while the original functions of **P**_**4**_, such as inhibition of EBNA1 dimerization and cytotoxicity to EBV-infected cells/tumors, are maintained.

#### L_2_P_4_-Based Zn^2+^ Binding Theranostic Agent ZRL_5_P_4_

A previous study indicated that Zn^2+^ is necessary for EBNA1 to dimerize and activate the *oriP*-enhanced transcription (50). Thus, we further modified the EBNA1-targeting peptide **P**_**4**_ by incorporating a zinc chelator (**ZRL5**) into the EBNA1-binding peptide **P**_**4**_, forming in **ZRL**_**5**_**P**_**4**_ (75). **ZRL**_**5**_**P**_**4**_ can respond independently to its interactions with Zn^2+^ and EBNA1 by emitting different fluorescence. **ZRL**_**5**_**P**_**4**_ was shown to strongly bind EBNA1 and to have specific *in vitro* and *in vivo* growth-suppressive activities in EBV-positive NPC cells.

Interestingly, **ZRL**_**5**_**P**_**4**_ could also selectively inhibit EBNA1 oligomerization, which occurs in the presence of Zn^2+^, while this new probe had little effect on dimer formation. That is, although **L**_**2**_**P**_**4**_ could completely suppress the dimerization in the absence of Zn^2+^, its suppression of dimerization was only partial when Zn^2+^ was present, indicating that Zn^2+^ can assist the dimerization. Indeed, it was suggested that the N-terminal UR1 domain in EBNA1 is the second dimerization site, via the coordination of Zn^2+^, beside the DBD (50). This is supported by our dot-blot binding assay showing that **ZRL**_**5**_**P**_**4**_ could interact with UR1 to disrupt the oligomerization (unpublished observation), whereas **L**_**2**_**P**_**4**_ showed no interaction and could not interfere with the multimerization, indicating that UR1 is responsible for a higher-order EBNA1 structure.

A fluorescent signal was emitted when **ZRL**_**5**_**P**_**4**_ bound with EBNA1, and its interaction with both UR1 and DBD might explain why **ZRL**_**5**_**P**_**4**_ could bind with EBNA1 and remain in the nuclei more than **L**_**2**_**P**_**4**_. Furthermore, **ZRL**_**5**_**P**_**4**_ can disrupt transactivation and induce EBV reactivation more potently than **L**_**2**_**P**_**4**_, suggesting that EBNA1 oligomers are more important than the dimer in some of EBNA1's functions (75). Importantly, we found that treatment with **ZRL**_**5**_**P**_**4**_ alone could reactivate EBV lytic induction by expressing the early and late EBV lytic genes and proteins. **ZRL**_**5**_**P**_**4**_ can also specifically elevate Dicer1 and PML expression, molecular events that have been reported to occur after the depletion of EBNA1 expression in EBV-infected cells (44, 76). Lytic induction is likely mediated by disruption of EBNA1 oligomerization and the subsequent change of Dicer1 expression.

To the best of our knowledge, **ZRL**_**5**_**P**_**4**_ represents the first specific agent to disrupt the EBNA1 protein and to potently reactivate EBV from latency, leading to tumor cell lysis and/or induction of viral proteins that presumably can be targeted by immune cells and antiviral agents to eliminate EBV-infected tumor cells. Importantly, this study also suggests the EBNA1 oligomerization is associated with the maintenance of EBV latency.

### EBNA1-Specific Gene Therapy

The consistent expression of EBNA1 in all latently infected cells is a unique feature of EBV-associated cancers, and has prompted researchers to investigate whether EBNA1-driven gene expression could be used as an EBV-specific targeted gene therapy for NPC. In 2002, Li et al. reported the first establishment of a recombinant adenovirus with wild-type p53 cloned downstream of the FR regions. Using this adenoviral vector, they successfully induced wild-type p53 expression in the EBV-positive NPC cell line C666-1 (77). The specific EBNA1-driven p53 expression retarded cell growth and induced apoptosis. Moreover, the combination of EBNA1-driven p53 expression and ionizing radiation decreased cancer-cell viability synergistically in both *in vitro* and *in vivo* NPC models, and the precise triggering of p53 expression on EBV-associated cells spared normal cells, leaving them unaffected.

The same research group has exploited such EBNA1-specific adenoviral vectors, confirming the utility of these constructs for inducing expression of BimS proapoptotic factor and FASL death ligand for effective treatment of NPC (78, 79). They have also generated a conditionally replicating adenovirus (CRA), adv.oriP.E1A, wherein E1A is expressed in an EBNA-1-dependent manner. Treatment of EBV-positive NPC cells with adv.oriP.E1A resulted in specific E1A expression and cytotoxicity, and combination of adv.oriP.E1A with ionizing radiation (i.e., RT) caused tumor regression in EBV-positive NPC xenografts and is associated with minimal systemic toxicity (80). Unfortunately, a study on the pharmacokinetics and biodistribution of EBV-specific transcriptionally targeted adenoviruses revealed that the vectors were mainly sequestered in the liver, limiting their potential clinical application. Nevertheless, strategies modifying the adenoviral vector have been shown to improve tumor uptake and reduce non-specific uptake, affording enhanced therapeutic efficacy (81).

In addition to adenovirus-based vectors, a novel minicircle non-viral vector, mc-oriP-IFNγ I, was developed to drive IFNγ expression by EBNA1 for effective targeting of EBV-positive tumor cells in NPC xenograft models via intratumoral injection (82). Similarly, an EBNA1-specific minicircle non-viral vector expressing *miR-31-5p* was constructed for EBV-specific targeted therapy. *miR-31* is a tumor suppressor microRNA commonly inactivated in NPC by homozygous deletion and promoter hypermethylation. Although mc-oriP-miR-31 inhibits cell proliferation and migration of C666-1 *in vitro* by EBV-specific induction of *miR-31, in vivo* studies on systemic delivery of this vector in NPC xenografts are needed to prove its therapeutic efficacy (83).

Meanwhile, the latent EBNA1 protein has also been exploited for oncolytic therapy via reactivation of the viral lytic cycle. Wang and co-workers (84) have reported EBV lytic reactivation in NPC cells by transfecting an EBNA1-driven CMV-BZLF1 expression plasmid (84). While the transfection of the CMV-driven BZLF1 plasmid induced moderate expression of BZLF1 in an EBV-infected epithelial cell line, the presence of an FR enhancer DNA element in the vector further promoted the induction of BZLF1, with BZLF1 triggering EBV reactivation, as shown by the expression of early and late viral genes, and causing in cell death. However, the vector could also induce mild expression of BZLF1 in EBV-negative cells, which may promote oncogenesis in patients' normal cells (6).

In addition to *BZLF1*, another immediate-early (IE) lytic gene, *BRLF1*, can also trigger EBV reactivation. Wang et al. have shown that EBNA1-driven BRLF1 expression induces EBV reactivation in C666-1 cells (85). A baculoviral vector with a *BRLF1* expression cassette cloned downstream of the EBV oriP enhancer element was designed to trigger lytic reactivation in various NPC cell lines. This EBNA1-driven BRLF1 viral vector was observed to cause lytic EBV DNA replication and cell death in infected tumor cells. Moreover, the baculovirus-infected NPC cells caused significant tumor-growth retardation in nude mice.

The above examples unambiguously suggest that EBNA1-FR interactions are a promising target for EBV-specific therapy. Nevertheless, the success of these EBNA1-specific gene therapeutic approaches is dependent on the efficiency of delivery of these vectors to the cancer cells. In this context, recent advances in non-viral delivery technologies mean that the antitumor effect of EBNA1-specific therapeutic constructs delivered by biocompatible nanoparticles in *in vivo* EBV-positive NPC models must also be investigated to confirm their utility in clinical applications.

### Inhibition of Latent Membrane Proteins

LMP1 is believed to be a viral oncoprotein promoting transformation and progression of NPC via activation of multiple cellular signaling pathways, such as the NF-κB, PI3K/AKT, MAPK, and IRF pathways (36). Although high LMP1 expression has been reported in ~25–30% of NPCs, a recent genomic study has revealed that this subgroup of tumors is characterized by a lack of somatic alterations for activating NF-κB signaling and other driver mutations (15). In low-LMP1-expressing tumor specimens, heterogeneous expression in a subpopulation of cancer cells was revealed by immunohistochemistry. As LMP1 has been shown to induce cancer stem/progenitor cells, the occurrence of such a subpopulation of LMP1-expressing cells may be important for maintaining the tumorigenic properties of NPC cells (37, 38). Thus, targeting LMP1 is hypothesized to be a potential therapeutic intervention for NPC, even in tumors exhibiting weak LMP1 expression. This hypothesis is supported by various LMP1-targeting studies in a native EBV-positive NPC cell line, C666-1, that weakly expresses the LMP1 protein. While several studies on LMP1 targeting have been conducted in preclinical models, interesting clinical trial results have also been reported in a cohort of NPC patients (86, 87).

Among various RNA-interference technologies, inhibition of LMP1 expression by use of an RNA-cleaving DNAzyme has been extensively explored as a potential therapeutic strategy for NPC. DNAzymes are synthetic, single-stranded catalytic DNA molecules with excellent stability and activity in downregulating gene expression. They can be engineered to bind to the complementary sequence of RNA according to the Watson-Crick model. Upon binding to the target mRNA sequence, the DNAzyme can mediate the cleavage of RNA molecules at purine:pyrimidine junctions (86).

The general structure of a DNAzyme consists of a catalytic domain of 15 deoxyribonucleotides, flanked by two substrate-recognition domains, each containing seven to nine deoxyribonucleotides. In 2005, Lu et al. reported the first successful identification of the conserved regions of LMP1 targeted by DNAzymes (88). They further demonstrated the ability of a sequence-specific DNAzyme to knockdown LMP1 gene expression, impair downstream NF-κB signaling, and induce apoptosis in B95.8 cells. Due to the lack of EBV-positive LMP1-expressing NPC cell lines, their subsequent studies on NPC have only examined the inhibitory effects of these DNAzymes on LMP1-transfected epithelial cells (87, 89, 90). Interestingly, Ke et al. (89) have shown that a specific LMP1-targeted DNAzyme, DZ509, inhibited cell proliferation and induced apoptosis in an EBV-positive NPC cell line C666-1 with weak LMP1 expression (89). Intratumoral injections of this DNAzyme also significantly suppressed tumor growth in nude mice models. The vulnerability of C666-1 cells to the inhibition of LMP1 may imply the importance of weak LMP1 expression in tumorigenesis, supporting the therapeutic implications of this novel LMP1-targeting approach.

A clinical trial to evaluate the therapeutic efficiency of an LMP1-targeting DNAzyme, DZ1, as a radiosensitizer was conducted in a cohort of 40 patients with LMP1-positive NPC (87). The study found that there was a lower short-term tumor regression rate and altered tumor vasculature in the DZ1 treatment group. Furthermore, no adverse outcomes of the combined RT and DZ1 treatment were observed. In these clinical studies, DZ1 or control saline was directly injected into tumors with the guide of an endoscope, but not systematic administration. The utility of LMP1-targeted DNAzyme as a systemic treatment for NPC patients, especially with advanced disease, needs to be further elucidated using efficient *in vivo* delivery vehicles, such as nanoparticles (90).

Aside from EBNA1 and LMP1, only a few other latent EBV gene products have been targeted in NPC. Although LMP2 serves as a major viral antigen for developing therapeutic vaccines and T-cell-based immunotherapies in NPC patients, the effects of targeting LMP2 on tumor suppression in native EBV-positive NPC cells are not well-defined. The heterogeneous expression of LMP2 in tumor specimens also suggests that the clinical benefits of this approach may be limited. However, the role of LMP2A in promoting cancer stem-cell properties suggests that targeting LMP2 is a potential therapeutic strategy for this EBV-associated epithelial cancer.

## Cytolytic Therapies Switching EBV Latency to Lytic Cycle

Cytolytic therapy utilizes a naturally occurring virus or genetically engineered virus that can selectively lytic replicate and kill the host cells, without harming normal cells (91). Distinct from the HBV- and HPV-associated cancers, in which integration of the viral genome occurs as a critical event during transformation, clonal EBV episomes are consistently found in EBV-associated malignant diseases (3, 6). This episomal nature of the EBV genome implies that induction of the viral lytic cycle could serve as a cytolytic therapy to cure NPC. When latent EBV are induced into the lytic cycle, the IE proteins BZLF1 and BRLF1 must be expressed, as these further activate the transcription of early and late proteins to progress continue the lytic infection cycle (92). Lytic replication in EBV-positive NPC cells will result in cell disruption, for the release of infectious viral particles.

**Figure 3:**
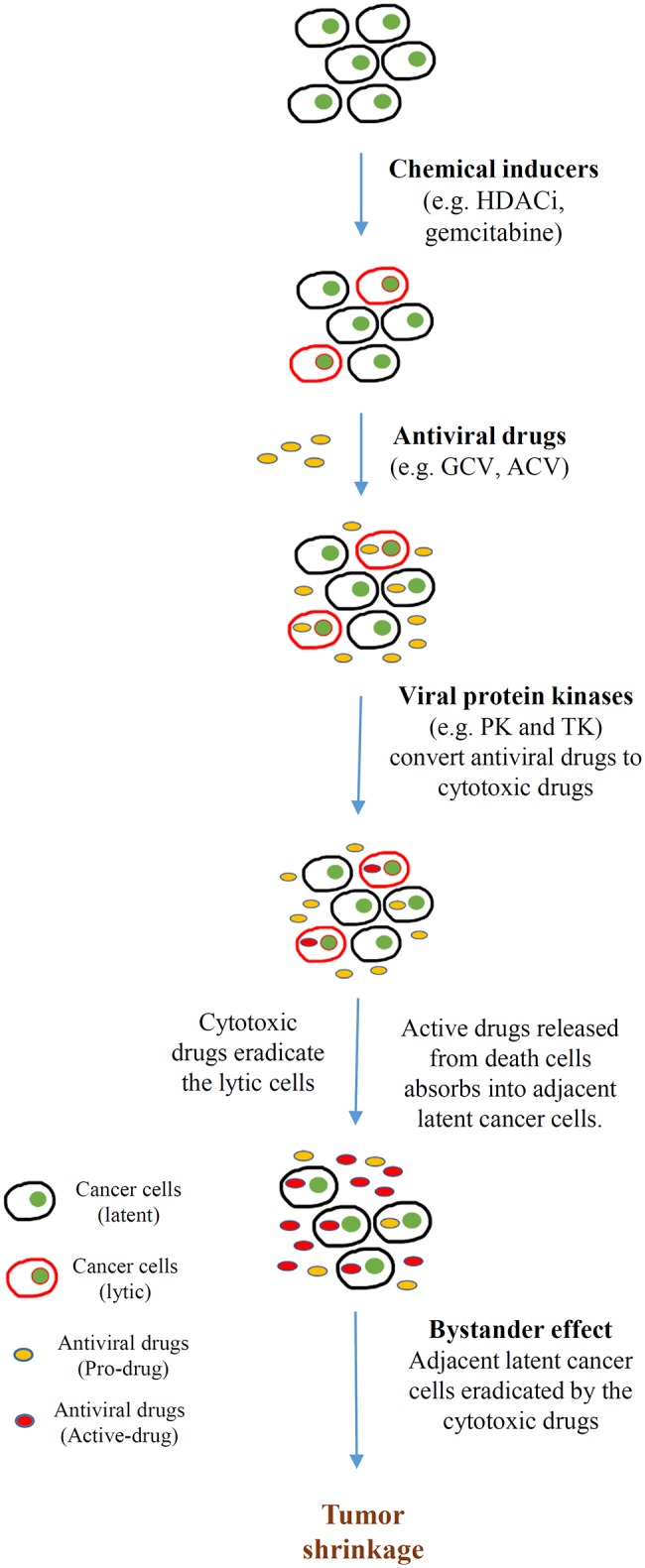
Cytolytic therapy of Epstein-Barr virus (EBV)-associated cancers. Cancer cells harboring EBV in latent infection are induced to undergo reactivation by different chemical inducers [e.g., histone deacetylase inhibitors (HDACis), DNA-damaging agents (gemcitabine)]. Subsequently, the tumor is treated with antiviral drugs [e.g., ganciclovir (GCV), acyclovir (ACV)] that are non-toxic unless converted from prodrugs to active drugs by a sequence of phosphorylation reactions. The monophosphorylated form of the antiviral drugs is first catalyzed by either BGLF4 (PK) or BXLF1 (TK), which are induced by the EBV immediate early (IE) genes BZLF1 and BRLF1. Subsequently, cellular kinases catalyze the formation of the cytotoxic diphosphate and triphosphate forms of the drugs. These cytotoxic drugs incorporate into the lytic cells, resulting in apoptosis. Apoptotic cells break down and release the toxic drugs to the tumor microenvironment. Adjacent latent cancer cells absorb the released drugs (the bystander effect) and are eradicated by them. This bystander effect then further promotes tumor shrinkage.

In addition to directly promoting cell death, lytic cycle induction can raise the potency of immune responses and induce susceptibility to antiviral agents in EBV-associated cancers. The early lytic proteins BGLF4 [EBV protein kinase (PK)] and BXLF1 [EBV thymidine kinase (TK)] are enzymes that can metabolize and activate prodrugs, such as ganciclovir (GCV), acyclovir (ACV), and fialuridine (FIAU). Although these antiviral agents have no effect on latently infected cells, phosphorylation of these prodrugs by EBV PK and TK during lytic reactivation induces premature termination of the nascent DNA and thus induces apoptosis, facilitating the cytotoxicity and bystander effect in the lytic and adjacent tumor cells (Figure 3) (92). By exploiting this unique feature, various approaches targeting the latent-lytic switch have been explored as potential therapeutic strategies for EBV-associated malignancies.

### Latent-Lytic Switch in EBV-Infected Cells

In lymphocytes, EBV establishes a life-long latency stage upon infection, while it frequently undergoes lytic replication in the epithelial cells of healthy carriers. This is consistent with the fact that primary EBV infection takes place at the oral epithelium, after which the lytic infection of oral epithelial cells triggers the release of infectious virions that infect the surrounding B lymphocytes (6, 17). Despite the tendency of EBV to undergo lytic infection in epithelial cells, lytic reactivation is rarely detected in EBV-positive NPC tumor cells. This shows that the latent-lytic switch in NPC cells is tightly regulated by both viral latent genes and cellular factors.

Epigenetic modification of the viral genome, cellular transcription repressors, and a number of EBV-encoded microRNAs have been shown to contribute to inhibiting lytic gene expression (93–96). In latently infected cells, global methylation of the EBV genome interferes with BZLF1- and BRLF1-driven early and late lytic gene transcription and suppresses spontaneous EBV reactivation (93, 94). An EBV microRNA, *miR-BART20-5p*, directly inhibits the expression of BZLF1 and BRLF1 to maintain latency in EBV-associated gastric cancer (95). As shown in our recent study, several abundantly expressed miR-BARTs (*miR-BART5-5p, BART7-3p, BART9-3p*, and *BART14-3p*) inhibit lytic reactivation via suppression of ATM expression in NPC cells (96). Furthermore, the transcription of the two IE genes, *BZLF1* and *BRLF1*, is regulated by multiple transcription repressors including YY1, E2-2, MEF-2D, and ZEB1/2 in EBV-infected cells (92). Extensive studies have also revealed that multiple cellular events, such as aberrant protein kinase C (PKC), TGF-β and other signaling pathways, cell differentiation, hypoxia, DNA damage, and reactive oxygen species (ROS) induction play key roles in EBV lytic reactivation in B cells and epithelial cells, by activating the promoters of *BZLF1* and *BRLF1* (92). Nevertheless, the effects of these cellular factors on lytic-cycle induction remain to be defined in EBV-positive NPC cells.

Notably, induction of BZLF1 or BRLF1 alone is sufficient to activate the lytic cycle in EBV latently infected cells (97). BZLF1 and BRLF1 proteins are able to activate both their own and one another's promoters, resulting in efficient lytic-cycle induction. BZLF1 preferentially activates lytic promoters that are methylated, whereas BRLF1 preferentially activates unmethylated lytic promoters (92, 93). BZLF1 induces the transcription of BRLF1 by binding directly to Z-responsive element (ZRE) DNA elements on the BRLF1 promoter (Rp). In contrast, BRLF1 indirectly binds to the BZLF1 promoter (Zp) via interaction with other cellular factors, forming a positive loop to drive their transcription. Thus, the ability of EBV to switch from latent to lytic infection is largely determined by the presence of cellular transcriptional activators that stimulate Zp or Rp, and the inactivation of cellular transcriptional repressors that simultaneously suppress Zp or Rp (92). Then, these two IE proteins (*BZLF1* and *BRLF1*) cooperatively activate the promoters of early lytic genes involved in viral genome replication.

EBV lytic replication is initiated at two replication origins, known as oriLyts, in the EBV genome. This is accompanied by the related EBV-encoded replication proteins BALF5 (DNA polymerase), BMRF1 (DNA polymerase processivity factor, also called diffuse early antigen EA-D), BBLF2 (single-stranded DNA-binding protein), BBLF4 (helicase), BSLF1 (primase), and BBLF2/3 (a component of the helicase-primase complex). The late genes encoding structural proteins (viral capsid antigen and gp350) are expressed after viral genome replication, to assist EBV virion production (97–99).

### Lytic Cycle-Inducing Agents

**Figure 4:**
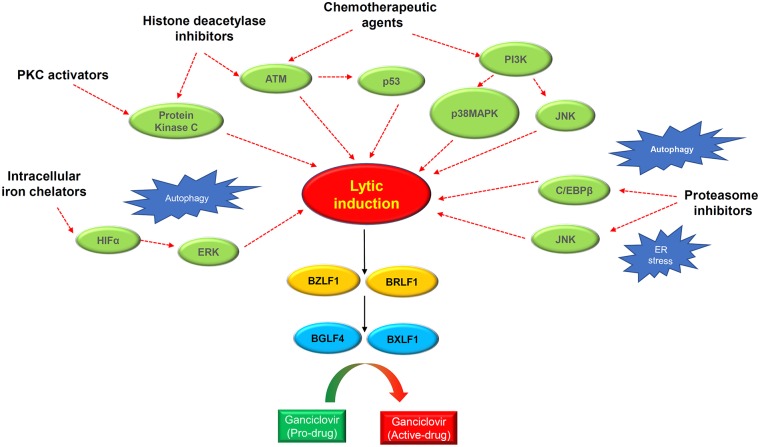
Schematic diagram showing the rationales of lytic induction treatment of EBV-associated cancers. Multiple classes of chemical inducer trigger EBV lytic induction via activating different cellular signaling pathways with extensive cross-talks. Histone deacetylase inhibitors and protein kinase C (PKC) activators induce the PKCdelta and ATM signaling pathway. Chemotherapeutic agents activate the ATM-p53 signaling axis as well as the PI3K/p38MAPK/JNK signaling. Proteasome inhibitors trigger autophagy and ER stress which induce EBV reactivation via activating JNK and C/EBPβ. The intracellular iron chelators induce hypoxia via HIFα and ERK activation, causing EBV lytic induction. Through inducing the IE genes *BZLF1* and *BRLF1*, the chemical inducers switch on EBV lytic cycle. The expression of IE proteins further induces the early lytic proteins BGLF4 (protein kinase) and BXLF1 (thymidine kinase) which convert the ganciclovir into cytotoxic drugs to kill cancer cells during the cytolytic treatment.

Lytic reactivation of EBV can be observed in a small number of tumor cells in clinical specimens, as well as in the newly established EBV-positive NPC PDXs and cell lines (25). This observation strongly supports the potential clinical application of lytic induction therapy in NPC patients. To this end, multiple preclinical and clinical studies have been conducted over the past two decades to explore various lytic cycle-inducing agents for cytolytic reactivation therapy in EBV-associated NPC. These lytic inducers include chemotherapeutic agents, phorbol esters, histone deacetylase inhibitors (HDACis), and a number of novel chemical compounds identified by large-scale screening studies (Figure 4, Table 2).

**Table 2:** Chemical agents that reactivate the Epstein-Barr virus (EBV) lytic cycle in nasopharyngeal carcinoma (NPC).

**Class of lytic inducers**	**Cell types**	**Mechanism of action**	**References**
**Conventional chemotherapeutics**			
Cisplatin (CDDP) 5-Fluorouracil (5-FU) Gemcitabine (GEM)	C17 xenograft C17 xenograft C666-1	DNA-damage agents cause inhibition of DNA replication by DNA intrastrand crosslinking (cisplatin), interference with base-excision DNA repair (gemcitabine), and interrupting thymidine synthesis by inhibiting thymidine synthase (5-FU)	(100) (100) (101)
**Histone deacetylase inhibitors**			
Suberoylanilide hydroxamic acid (SAHA) Valproic acid (VPA) Sodium butyrate (SB) Trichostatin A (TSA) Romidepsin	C666-1 C666-1 NA, HA NPC-TW01-NA; NPC-TW04-4A HA and C6661	Inhibition of deacetylation of histones causes chromatin decondensation and interferes with gene transcription	(102, 103) (104) (104) (104, 105) (106)
**Protein kinase C (PKC) activators**			
12-*O*-tetradecanoylphorbol-13-acetate (TPA)	NPC43	Activation of the PKC signaling pathway	(25)
**DNA demethylating agents**			
5-Azacytidine	Eight patients with NPC	Inhibition of DNA methyltransferase causing hypomethylation of DNA, restoration of gene expression	(107)
**Intracellular ion chelators**			
C7	C666-1, NPC43, HA, HONE1-EBV	Chemical compound contains a metal-binding moiety that chelates Fe^2+^ and results in activation of autophagy	(108–110)
**Proteasome inhibitors**			
Bortezomib	HA	Proteasome inhibitor binds to the catalytic site of the 26S proteasome, resulting in inhibition of protein degradation via the ubiquitin-mediated proteasome degradation pathway	(102)
**ROS-related chemicals**			
*N*-methyl-*N*′-nitro-*N*-nitrosoguanidine (MNNG)	NA, HA, and C6661	*N*-nitrosoguanidine results in ROS production, inducing EBV reactivation through a p53-dependent mechanism	(111, 112)
**Other small synthetic organic compounds**			
E11/E7/C8/A10	C6661 and HONE1-EBV	Nil	(108)
**Antibacterial agents**			
Clofoctol	C666-1	Activation of the unfolded protein response (UPR), which is a stress-signaling pathway that extends from the endoplasmic reticulum (ER) to the nucleus through the PERK-eIF2α-ATF4-CHOP axis (16)	(113)

*ROS, reactive oxygen species*.

#### Chemotherapeutic Agents

Several FDA-approved drugs for chemotherapy show cell-context-specific ability to switch latency to the lytic cycle in EBV-infected cells. The EBV lytic reactivation ability of these chemotherapeutic agents may contribute to the chemosensitivity of EBV-associated NPC. Two chemotherapeutic agents commonly used in NPC treatment, cisplatin and 5-fluorouracil (5-FU), were able to induce lytic reactivation and confer GCV susceptibility in the EBV-infected gastric cancer cell line AGS-EBV and an EBV-positive NPC PDX model, C18 (100). The activation of IE and early lytic genes by these drugs was found to be dependent on the MAPK/ERK, p38 MAPK, and PKCδ signaling pathways. Meanwhile, gemcitabine is also an effective chemotherapeutic agent in NPC patients, and reactivates BZLF1 expression in the EBV-positive NPC cell C666-1 (101). Gemcitabine induced functional EBV-lytic proteins BGLF4 (PK) and BXLF1(TK) were found in an EBV-associated gastric cancer (EBVaGC) mouse model by [125I]-FIAU-based single-photon emission computed tomography (SPECT) planar imaging. Notably, the induction of the EBV lytic cycle by gemcitabine is mediated by activation of the ATM/p53 genotoxic stress pathway (114).

Cytolytic viral activation (CLVA) therapy using a combination of gemcitabine, the HDACi valproic acid (VPA), and GCV has been developed as a novel NPC treatment. The combination of gemcitabine and VPA showed a synergistic effect on inducing expression of EBV lytic gene expression, while inclusion of GCV further enhanced the cytotoxicity in the tumor cells. In preclinical studies, the treatment induced the EBV lytic cycle and exerted cytotoxicity in both *in vitro* and *in vivo* EBV-positive NPC models (101, 115, 116). A pilot clinical study of CLVA therapy revealed that the treatment was well-tolerated and resulted in disease stabilization and improved quality of life in three patients with progressive end-stage NPC (101). Although the safety of the treatment was confirmed in a subsequent Phase I/II study on eight patients with recurrent or metastatic tumors, only partial clinical response and stable disease were observed, in two and three of the patients, respectively (117). These findings show that clinical benefit of CLVA treatment needs further evaluation in large-scale clinical studies that include a patient group receiving gemcitabine treatment only.

#### Histone Deacetylase Inhibitors

Histone acetylation is a reversible posttranslational modification that modifies chromatin structure, changing the accessibility of transcription activators and/or repressors to the gene promoters. The gross change of histone acetylation status in a particular gene locus is due to the respective activities of histone deacetylases (HDACs) and histone acetyltransferases (HATs) (118): HDACs deacetylate histones and other non-histone proteins, while HATs catalyze the transfer of acetyl groups from acetyl coenzyme A to the lysine residues of proteins. Both act as cofactors of different transcription regulators for modifying chromatin structure. To date, a variety of FDA-approved HDACis with high activity have been discovered (119). The HDACis are categorized into five groups according to their structure: that is, cyclic peptides, hydroxamic acids, benzamides, short-chain fatty acids, and sirtuin inhibitors (119).

In view of the chromatin-like structure of the EBV episome, the therapeutic potential of various classes of HDACi have been studied, aiming to reactivate the silenced EBV IE genes in the EBV-associated malignancies (4). Among the HDACis investigated in EBV-positive epithelial cancer cell lines, suberoylanilide hydroxamic acid (SAHA), alone or in combination with a proteasome inhibitor (bortezomib), was found to be superior in terms of inducing EBV reactivation and causing cancer cell death (102, 120). As a class I HDACi, SAHA inhibits histone acetylase classes I and II via chelating the cofactor Zn^2+^ ion.

Notably, the combined treatment of bortezomib and SAHA enhanced ROS production, which caused cell apoptosis and at the same time suppressed virion production (121, 122). Concomitantly, ROS is proposed to be the intermediates of oxidative stress that mediate the EBV reactivation. A novel signaling mechanism in which ROS induces EBV reactivation was proposed by Huang and colleagues, based on the fact that ROS activates multiple signaling pathways, such as ATM, p38 MAPK, and JNKs, to induce p53-dependent EBV reactivation (111). Moreover, oxidative stress has been found to initiate BZLF1 transcription in the Raji cell line (123). These results indicate that the DNA damage response and ROS play a direct role in triggering EBV reactivation in response to various lytic induction treatments.

Although HDACi treatments are effective in triggering EBV reactivation in particular cell line models, their broad efficacy in cell line models remains in doubt, as varied ability of HDACis to trigger EBV reactivation in different cell lines has been highlighted recently (121). Moreover, the underlying mechanisms of the induction of IE genes by these treatments have generally not been investigated and therefore remain elusive. Although it is believed that the opened chromatin structures of IE promoters are critical for their induction, gene transcription does not occur efficiently unless transcription repressors are displaced from the IE promoters. Furthermore, SAHA is known to alter the acetylation of non-histone proteins as well (122). Thus, it is conceivable that understanding the acetylation status of other non-histone proteins will be critical to solve the discrepancy between different studies using HDACis.

#### Protein Kinase C (PKC) Activators

Early studies revealed that the phorbol ester 12-*O*-tetradecanoylphorbol-13-acetate (TPA) reactivates the EBV lytic cycle in various EBV-infected lymphoid cells through activation of the PKC signaling pathway (124, 125). Later, Gao and colleagues defined the mechanism of EBV lytic induction by TPA in epithelial cells. In EBV-infected gastric cell lines, they showed that TPA activates PKC and MAPK signaling to mediate the increased binding of NF-κB and AP-1 to the BZLF1 promoter for inducing EBV reactivation (126).

The involvement of PKC-delta activation in the EBV latent-lytic switch has also been shown in NPC cells treated with HDACi and the microtubule-depolymerizing agent nocodazole (127, 128). Although TPA alone can induce the lytic cycle in EBV-positive NPC cells in a cell-context-dependent manner, a combination of TPA and other HDACi may be needed to maximize the induction of EBV reactivation (25, 104). As TPA is a classical tumor-promoting agent and can cause skin carcinogenesis (129), it is intrinsically unsuitable as a clinical drug. Nevertheless, other clinically approved PKC activators are worth exploring for their potential ability to induce the lytic cycle in NPC cells.

#### Intracellular Iron Chelators

Using the recombinant EBV-infected epithelial cancer cell lines AGS-BX1 and NA, Choi and colleagues performed a high-throughput phenotypic screening of 50,240 novel small organic compounds for chemical inducers of the EBV lytic cycle (108). Five compounds showed dose-dependent induction of EBV lytic genes at micromolar concentrations and specific cytotoxicity in EBV-infected epithelial cells. While these compounds were structurally diverse and distinct from classical lytic inducers such as phorbol esters or HDACis, one of the novel compounds, C7, was demonstrated to induce the EBV lytic cycle in multiple native EBV-infected epithelial cancer cells, such as C666-1, SNU-719, and YCCEL1. In addition, a recent study has shown that combined treatment with C7 and an HDACi resulted in apoptosis-mediated synergistic killing of EBV-positive NPC cells (109).

As C7 contains a metal-binding moiety and functions as a chelator of intracellular iron, the group has further examined the ability of other iron chelators, such as Dp44mT, deferoxamine, deferiprone, and deferasirox, to reactivate the EBV lytic cycle in EBV-positive epithelial cancers. Their functional study revealed that C7 and the other iron chelators reactivate the EBV lytic cycle through the chelation of intracellular iron, resulting in HIF-1α induction, ERK1/2 activation, and initiation of autophagy. Thus, iron chelators appear to activate hypoxic signaling and autophagy to trigger lytic reactivation in EBV-positive epithelial cancer cells (110). The above studies have identified the clinically available iron chelators as a novel class of lytic inducer for potential cytolytic therapy.

### Future Aspects

In the past decades, the development of EBV lytic-induction treatment for NPC has been hampered by the limited availability of patient-derived EBV-positive NPC models. Aside from the native EBV-positive NPC cell line C666-1, most of the preclinical studies on the efficacy of lytic inducers in reactivation of the EBV lytic cycle have been conducted on EBV-reinfected epithelial cells (e.g., EBV-CNE1, HA, HONE1-EBV) with undefined genome backgrounds. Due to the importance of cellular factors in the regulation of the latent-lytic switch in EBV, the response of NPC cells to different classes of lytic inducers is believed to be cell-context-specific. Specifically, NPC cells in each individual tumor might evolve and acquire different somatic changes to ensure EBV latency during cancer progression. This is evidenced by recent Phase I/II trials of CLVA treatment, in which the induction of the lytic cycle by gemcitabine and VPA appeared to vary among patients. This is itself consistent with preclinical findings that the combination of gemcitabine and VPA fails to induce the lytic cycle in some EBV-positive NPC cell lines, e.g., NPC43 and C17 (unpublished observation).

Furthermore, different efficiencies of HDACis or PKC activators in activating EBV lytic genes were observed in EBV-positive NPC cell lines (e.g., C666-1, NPC43, and C17) (25, 102–104). Recent genomic studies have also revealed that a number of somatic alterations (e.g., *TP53* and *TGFBR2* mutations) may contribute to the maintenance of EBV latency and regulation of the latent-lytic switch in NPC cells (15, 16, 130). Somatic mutations of *TGFBR2* may prevent NPC cell differentiation, which is a key cellular factor for reactivation of the EBV lytic cycle. The important roles of the DNA damage response and ROS induction in EBV reactivation imply that the status of *TP53* mutations in NPC cells may determine their response to lytic-inducer treatment.

The use of more genomically characterized patient-derived NPC models and native EBV-infected cell lines in comprehensive studies on the association of somatic genetic changes in these tumors with their response to different classes of chemical inducer may allow us to develop effective cytolytic treatment strategies for NPC patients (24, 25). In addition, uncovering the cellular mechanisms of the resistance of tumor cells to lytic cycle induction is important for improving the efficacy of this treatment. As reported previously, the constitutive activation of the NF-κB, STAT3, and WNT signaling pathways modulates major cellular mechanisms and viral gene expression during NPC tumorigenesis (3, 6). However, the roles of these NPC-associated oncogenic-signaling pathways in the switching of persistent latent infection to the lytic cycle have not been defined. The combination treatment of EBV lytic inducers with selected targeted inhibitors of these specific pathways in EBV-positive NPC tumors may provide new insights on the cellular signals that regulate lytic reactivation.

Finally, the high expression of multiple immunogenic viral lytic antigens during EBV lytic reactivation is expected to raise potent immune responses during cytolytic therapy. However, the activation of multiple immunomodulatory lytic proteins such as BNLF2a, BILF1, BGLF5, BCLF1, and BARF1 may dampen the treatment efficiency (131). BNLF2a, BILF1, BGLF5, and BCLF1 deregulate the host antigen-processing pathway via targeting TAP1 (peptide transporter associated with antigen processing 1) and HLA (human leucocyte antigen) class 1 molecules, thereby evading elimination of EBV lytic infected cells by host cytotoxic T lymphocyte (CTL) responses. Furthermore, BCLF1 and BARF1 function as immunosuppressive cytokine and antagonist of M-CSF, respectively. In addition to reduce natural killer (NK) and CTL responses, these lytic proteins suppress T cell activity through inhibition of IFN-γ. Notably, somatic alterations of HLA class 1 molecules (HLA-A, HLA-B, HLA-C) and their transcription regulator (NLRC5) were reported in 30% of NPC in which the immune responses to the viral lytic antigens are impaired (15). It is interested to elucidate the effect of cytolytic therapy on the host immune surveillance by comprehensively characterizing the tumor microenvironment in NPC humanized mouse models. The findings will enhance our outstanding of the host immune response to EBV targeting treatment.

## Author Contributions

All authors listed have made a substantial, direct and intellectual contribution to the work, and approved it for publication.

## Conflict of Interest

The authors declare that the research was conducted in the absence of any commercial or financial relationships that could be construed as a potential conflict of interest.
